# *Mycobacterium abscessus*—Bronchial Epithelial Cells Cross-Talk Through Type I Interferon Signaling

**DOI:** 10.3389/fimmu.2019.02888

**Published:** 2019-12-09

**Authors:** Chongxu Zhang, Huda Asif, Gregory E. Holt, Anthony J. Griswold, Michael Campos, Pablo Bejarano, Nevis L. Fregien, Mehdi Mirsaeidi

**Affiliations:** ^1^Section of Pulmonary, Miami VA Healthcare System, Miami, FL, United States; ^2^Division of Pulmonary and Critical Care, University of Miami, Miami, FL, United States; ^3^School of Medicine, John P. Hussman Institute for Human Genomics, University of Miami, Miami, FL, United States; ^4^Department of Pathology, Cleveland Clinic, Weston, FL, United States; ^5^School of Medicine, Department of Cell Biology, University of Miami, Miami, FL, United States

**Keywords:** *Mycobacterium abscessus*, bronchial epithelial cells, IFN, Interferon, mycobacteria

## Abstract

**Introduction:** *Mycobacteria* are aerobic non-motile organisms with lipid rich, hydrophobic cell walls that render them resistant to antibiotics. While there are over 150 different species of NTM, *Mycobacterium avium complex* (MAC) and *Mycobacterium abscessus* (MAB) are two of the most common culprits of pulmonary infection. MAB has been found to be most common in southeastern United States (Florida to Texas) and the third most rapidly growing NTM infection. It is responsible for chronic lung infections. Mycobacterial cell wall components initiate the interaction between bacteria and host. The reaction between bronchial epithelia and components in the envelope of mycobacterial cell wall is poorly understood.

**Methods:** A lung-on-membrane model was developed with normal human bronchial epithelial (NHBE) cells re-differentiated at the air-liquid interface (ALI) and human endothelial cells on a transwell^®^ polyester membrane. Microparticles from MAB cell walls were developed by an inhouse protocol and added to the ALI side of lung model. NHBE cells were harvested at day 3. RNA was isolated and analyzed with RNASeq. NHBE cells were lysed and protein assay was performed with western blot. We tested whether lung INF-alpha expression would increase in mice treated with intratracheal MAB cell wall particles. A paired *t*-test is used to compare two population means using GraphPad Prism 7 software.

**Results:** RNAseq analysis identified 1759 differentially expressed genes between NHBE cells challenged with and without MAB microparticles with FDR < 0.5. 410 genes had a 2.5-fold change (FC) or greater. NHBE cells exposure to MAB microparticles significantly enriched the IFN I signaling pathway. Protein overexpression of IFN I family (2′-5′-Oligoadenylate Synthetase 1, Interferon-induced GTP-binding protein Mx1, Interferon-stimulated gene 15) was found in bronchial epithelial cells following exposure to MAB cell wall microparticles. IFN-α protein and gene expressions were significantly increased in mice lung challenged with microparticles in comparison with controls.

**Conclusion:** These data strongly support the role of Type I IFN in cross-talk between NHBE cells and MAB. They also suggest that initiating immune response by NHBE cells may play a central role in innate immunity. Furthermore, this study underscores the importance of mycobacterial cell wall in initiating innate immune response.

## Introduction

Non-tuberculous mycobacteria (NTM) are ubiquitous organisms responsible for clinically significant lung infections that have increased 5–10% annually over the past two decades with an annual burden of ~84,000 cases (1). In the United States, *Mycobacterium Avium Complex* (MAC) is the most frequently isolated species followed by *Mycobacterium kansasii* and *Mycobacterium abscessus* (MAB) (2, 3). MAB is the most challenging NTM to treat due to high antibiotic resistance rates (4).

Mycobacterial cell walls contain multiple peptidoglycans including D-glucosamine and a mycolic acid layer (5) that initiate the interaction between bacteria and host upon inhalation (6). Macrophages are a critical immune cell in combatting mycobacterial infections with a significant proportion of their response dependent on type I IFN signaling (7, 8). However, the response of bronchial epithelial cells to mycobacterial infection is not well-described. Normal human bronchial epithelial (NHBE) cells express type I IFN that suppress viral replication, induce apoptosis and enhance Th1 immunity (9). NHBE cells exposed to MAB are known to upregulate expression of cytokine transcripts (10). We hypothesize that NHBE cells play a vital role in initiating the host response to MAB through production of pro-inflammatory type I IFN cytokines. To determine the effects of MAB exposure on NHBE production of type I IFN signaling, we investigated the gene expression profile, and protein expression changes in NHBE cell cultures. The immunologic effects of MAB-cell wall microparticles in lung bronchial and immune cells were tested in a mouse model.

## Methods

### Lung-on-Membrane Model (LOMM)

Our dual chamber lung model contains normal human bronchial epithelial (NHBE) cells re-differentiated at the air-liquid interface (ALI) on one side and human endothelial cells (Human Lung Microvascular Endothelial Cells, Lonza, Walkersville, MD) on the other side of a transwell^®^ polyester membrane cell culture inserts (12 mm diameter, 0.4 μm pore size; Corning Life Sciences, Amsterdam, The Netherlands). NHBE cells were collected from lungs rejected for transplant at University of Miami where epithelial cells were isolated from upper bronchi and cultured as previously reported (11–13). Both sides of the membrane were coated with collagen IV from human placenta (Millipore Sigma, St. Louis, MO, USA). 5 × 10^5^ NHBE cells were cultured on top of the membrane in bronchial epithelial cell growth medium (BEGM) until cells were confluent. The cells were placed on air and fed with ALI Media from bottom chamber thereafter. When NHBE cells were fully differentiated and became ciliated, 2 × 10^5^ endothelial cells were plated on the opposite side of the transwell membrane when membrane was upside down. The upside-down membrane was placed into humidified incubator at 37°C, 5% CO_2_ for 8 h to let endothelial cells to adhere. The transwell was flipped to the original position and both cells lines were feed with a 50:50 mixture of endothelial and epithelia cell media in the bottom chamber and were incubated for 24 h. NHBE cells were washed and the media was changed every 2 days. Two days after adding the endothelial cells, the lung model was used for experiment and the media was changed every 2 days. This lung model has been previously published (14). For the current study, primary NHBE cells from five individuals were used to develop LOMM. Table 1 shows demographic data and smoking history of lung donors.

**Table 1:** Shows age, race, and smoking history of lung donors.

**Subjects**	**Age**	**Race**	**Smoking**
1	60–65	European American	NS
2	65–70	Latino	NS
3	75–80	European American	NS
4	20–25	Latino	NS
5	35–40	European American	NS

### MAB Microparticle Production

MAB cell wall microparticles were isolated from a strain of MAB with a rough colony isolated from the sputum of an 11-year old boy with cystic fibrosis (isolate # CCUG 47942, gift from Dr. Malin Ridell, University of Gothenburg, Sweden). MAB is grown in Middlebrook 7H9 broth with ADC enrichment medium (Millipore Sigma, St. Louis, MO, USA) at 37°C. When the culture OD600 reached 1.0–1.2, cells were collected by centrifugation at 4,000 g for 10 min, washed once in PBS, centrifuged, resuspended using a 15:1 (volume to volume) ratio of lysis buffer, sonicated and incubated on ice for 30 min. The lysis buffer contains 137 mM sodium chloride, 10 mM sodium phosphate, 2.7 mM potassium chloride, and detergents and protease inhibitors. Lysed cell samples were then centrifuged at 3,000 g for 5 min to remove intact MAB cells. The supernatant was transferred to a new tube and centrifuged for 20 min. Twenty milliliters of fresh lysis buffer was and the pellet was resuspended by brief sonication and centrifuged at 12,000 g for another 20 min. The pellet was resuspended in 20 ml volume of PBS and kept at 95°C for 15 min. After cooling to room temperature, the lysate was centrifuged at 12,000 g for 20 min and the pellet was washed with PBS buffer 3 times at 12,000 g for 10 min. Finally, the pellet is suspended in Dulbecco's Modified Eagle Medium (DMEM) and stored at −80°C. The concentration of the microparticles is calculated by the following equation: Final concentration = Volume of original culture × OD600 × (2.2 × 10^8^ bacteria/ml)/final volume. High quality images of MAB particles were obtained by scanning electron microscope (SEM) and proven to be non-infectious by absence of growth of MAB in culture.

### Exposure of Epithelial Cells to MAB Microparticles

LOMM (bronchial epithelial cells side) were exposed to 100 μL of MAB microparticles diluted to a concentration equal to a multiplicity of infection (whole bacterium) of 10:1. Bronchial epithelial cells were harvested 72 h after exposure.

### Mouse Model Exposure to MAB Microparticles

We used 6-week-old age C57Bl/6 male mice purchased from the Jackson Laboratory (Bar Harbor, ME) in experiments approved by the Animal Studies Subcommittee (IACUC) at the Miami VA Health system. Individual mice were challenged intratracheally every 3 days with MAB microparticles for 4 doses using a 20 G angiocatheter inserted into the trachea. After tube placement, microparticles were injected with the first dose injecting 50 μL (~5 × 10^8^ CFU) and next three doses receiving 20 μL (~2 × 10^8^ of CFU). The control group received equivalent volumes of PBS intratracheally. Figure S1 shows the procedure and Figure S2 shows proof of correct instillation within the lung with use of methylene blue instillation.

Mice were sacrificed on day 14 and the left lungs were harvested for pathology after perfusion to remove blood. Lungs were filled with 10% buffered formalin and fixed in formalin for at least 72 h before immunohistochemistry (IHC) staining. H&E staining was used to determine inflammatory pathology. Lungs were stained with antibodies against CD4 (rabbit, Abcam, catalogue# ab133616), CD8 (rabbit, Abcam, #ab12512), CD68 (rabbit, Abcam, #ab12512), PD-L1(rabbit, Proteintech, #17952-1-AP), and IFN-α (rabbit, Abcam, #ab193055) antibodies to identify infiltrating immune cells. Lung inflammation was scored using the three fields with the highest infiltrate's intensity at 100X power magnification. The area of inflammation was measured and averaged for the three examined high power fields. The right lungs were removed and frozen at −80°C for later protein extraction and western blot analysis. Protein extracted from lung tissue was performed as previously described (15).

### RNAseq and Pathway Analysis

Total RNA from NHBE cells was extracted by using a Direct-zol™ RNA MicroPrep kit (R2060, Zymo Research Zymo Research, Irvine, CA), following the manufacturer's protocol. Briefly, cells were washed with PBS, lysed in TRIreagent and RNA was purified using a Direct-zol RNA column. DNase I treatment was performed on the column and RNA was eluted in DNase/Rnase Free water.

RNA from mouse lungs were extracted using RNA Miniprep Plus Kit (Zymo Research). Briefly, whole lung was homogenized in TRI reagent and total RNA extraction was performed following the instructions provided by the manufacturer with additional DNase treatment. Quantity and quality of the samples was determined by NanoDrop spectrophotometer and Agilent Bioanalyzer 2100, respectively. Samples with RNA integrity number > 8 were used for the analysis.

Preparation and sequencing of RNA libraries was performed at the John P. Hussman Institute for Human Genomics Center for Genome Technology. Briefly, total RNA quantity and quality were determined using the Agilent Bioanalyzer. At least 300 ng of total RNA was used as input for the KAPA RNA HyperPrep Kit with RiboErase (HMR) according to manufacturer's protocol to create ribosomal RNA-depleted sequencing libraries. Sequencing was performed on the Illumina NextSeq 500 generating ~40 million single-end 75 base reads per sample. Sequencing data were processed with a bioinformatics pipeline including quality control, alignment to the hg19 human reference genome, and gene quantification. Count data was inputted into edgeR software (16) for differential expression analysis. Counts were normalized using the trimmed mean of M-values (TMM) method (17) to account for compositional difference between the libraries and paired differential expression analysis using a generalized linear model with sample as a blocking factor. Genes were considered statistically different with a false discovery rate *p*-value (FDR) ≤0.05.

Pathway enrichment analyses was performed using Enrichr online (18) and DAVID bioinformatics resource (19) to obtain the enriched biological processes (BPs) and pathways with genes with a linear fold change (FC) >2.5.

### Western Blotting

NHBE cells and lung tissue cells were lysed in lysis buffer (Cell Signaling Technology, Beverly, MA) with protease inhibitor cocktail (Cell Signaling Technology, Beverly, MA and sonicated three times for 2 s each with at least 1-min rest on ice between each 2-s pulse. Samples were centrifuged at 10,000 × g for 5 min at 4°C and the supernatant was collected. Protein concentration was determined by BCA protein assay kit from Cell Signaling Technology.

Thirty micrograms of total protein were mixed in a reducing sample buffer, and then electrophoresed on a 10–15% Tris gel with Tris running buffer, blotted to PVDF membrane, and sequentially probed with primary antibodies against 2′-5′-Oligoadenylate Synthetase 1 (OAS1), Interferon-induced GTP-binding protein Mx1 (MX1), Interferon-stimulated gene 15 (ISG15) (Proteintech Group, Inc. Rosemont, IL). A horseradish peroxidase-conjugated goat anti-rabbit antibody was then added, and secondary antibodies were detected using enhanced chemiluminescence (ECL Plus, General Electric Healthcare, and Milwaukee, WI).

### Statistical Analysis

A paired *t*-test is used to compare two population means using GraphPad Prism 7 software. Results with *p* < 0.05 were defined as statistically significant.

## Results

### IFN I Signaling Pathway Genes Are Overexpressed in NHBE Cells Following MAB Exposure

**Figure 1:**
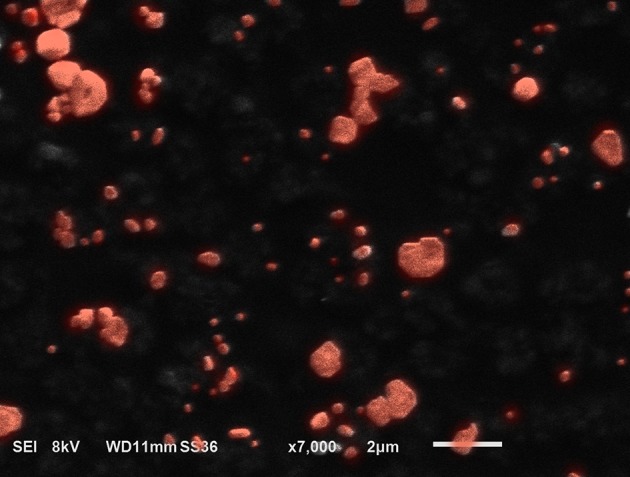
SEM image from microparticles developed from MAB. The particles are submicron to 2 microns in size.

**Figure 2:**
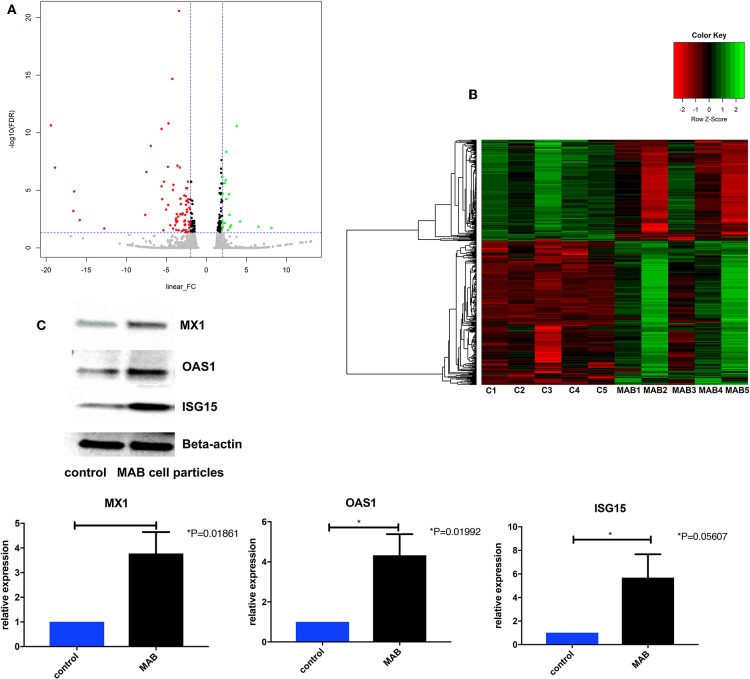
**(A)** Volcano plots differentiating genes of NHBE cells with and without exposure to MAB microparticles. **(B)** Heat map of RNA-Seq transcriptome analysis of bronchial epithelial cells with and without exposure to MAB. Genes that were identified as significantly different between two groups with a >2.5-fold increase (green) or decrease (red) in expression level (sample size was 5 for each group). **(C,D)** Representative western blot analysis of MX1, OAS1, and ISG15 expression in bronchial epithelial cells exposed to MAB cell wall particles.

The MAB cell wall particles (Figure 1) with a size that ranged from less than a sub-micron to 2 μm were exposed to NHBE cells and RNA and protein expression was analyzed. RNAseq analysis identified 1759 differentially expressed genes between NHBE cells challenged with and without MAB microparticles (FDR <0.5) and found 410 genes had at least a 2.5-fold change (FC). Volcano plots show marked differences in gene expression between NHBE cells with and without exposure to MAB microparticles (Figure 2A). Figure 2B shows the heatmap for unsupervised clustering of the RNAseq transcriptomes according to pearson correlation. Individual gene expression was normalized across samples to percentages ranging from marked downregulation (deep red) to marked upregulation (deep green).

The pathway enrichment analysis for gene differentially expressed 2.5 fold between NHBE cells with and without exposure to MAB microparticles. NHBE cells exposure to MAB microparticles significantly enriched the IFN I signaling pathway (GO:0060337) and cellular response to type I IFN (GO:0071357) (adjusted *p* = 0.00001047, and *p* = 0.00001047 respectively) in pathway analysis (Table 2). The top upregulated genes from the IFN I family (with FC>2.5 and FDR <0.5) were Radical S-Adenosyl Methionine Domain Containing (RSAD2) (FC 6.67), Myxovirus resistance 2 (MX2) (FC 5.66), Interferon induced protein 44 like (IFI44L) (FC 4.34), Interferon stimulated gene (ISG)15 (FC 4.34), Interferon Induced Protein With Tetratricopeptide Repeats 1 (IFIT1) (FC 4.20), Interferon Alpha Inducible Protein 6 (IFI) (FC 3.66), MX1 (FC 3.1), 2′-5′-Oligoadenylate Synthetase (OAS)1 (FC 2.79), and OAS3 (FC 2.69). Figures 2C,D show confirmation of increased protein expression of MX1, OSA1, and ISG15 using western blot in cultures exposed to MAB microparticles.

**Table 2:** The pathway enrichment analysis for gene differentially expressed 2.5 fold between NHBE cells with and without exposure to MAB microparticles.

**Index**	**Name**	***P*-value**	**Adjusted *p*-value**
1	Epidermal cell differentiation (GO:0009913)	8.304e-13	9.309e-10
2	Peptide cross-linking (GO:0018149)	5.967e-12	2.230e-9
3	Type I interferon signaling pathway (GO:0060337)	6.541e-8	0.00001047
4	Keratinocyte differentiation (GO:0030216)	3.633e-12	2.037e-9
5	Epidermis development (GO:0008544)	2.621e-9	5.876e-7
6	Regulation of nuclease activity (GO:0032069)	0.00005144	0.004436
7	Skin development (GO:0043588)	9.947e-10	2.788e-7
8	Negative regulation of viral genome replication (GO:0045071)	1.165e-7	0.00001632
9	Cellular response to type I interferon (GO:0071357)	6.541e-8	0.00001047
10	Negative regulation of viral life cycle (GO:1903901)	5.545e-7	0.00006907

### Overexpression of Cytokine Genes in NHBE Cells Following MAB Exposure

Cytokine genes expression profile of NHBE cells following exposure to MAB cell wall microparticles also showed significant upregulation of IL36β (FC 41.3), IL36α (FC 18.4), IL36γ (FC 3.2), IL 23A (FC 3.2), IL1RL1 (FC 3.1), IL1RN (FC 3.1), and IL1RN (FC 2.6). Chemokine profiles showed significant expressions of CCL5 (FC 8.8), CXCL11 (FC 3.1), CCL22 (FC 2.8), and CXCL10 (FC 2.5). We also found Matrix Metallopeptidase (MMP) 9 (FC 4) was differentially expressed between two groups.

### Granulomatous Reaction in the Lungs Following Exposure to MAB

**Figure 3:**
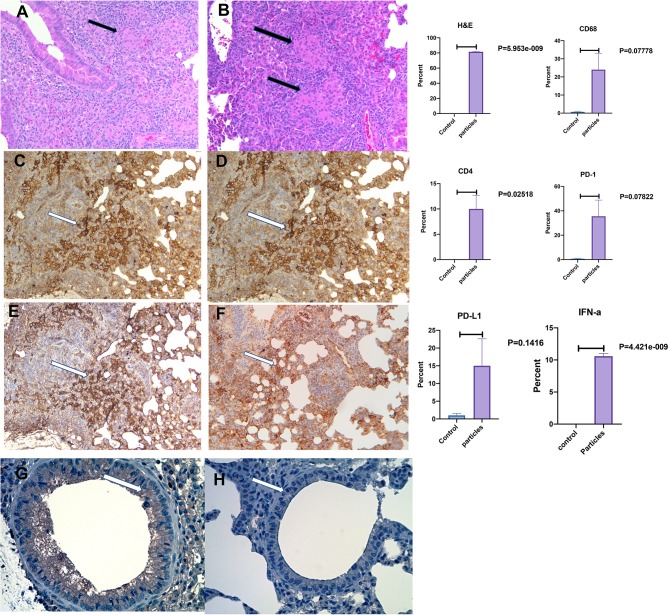
Development of non-caseating granuloma in the mouse lung after MAB microparticle challenge. **(A,B)** Black arrows show granulomas in the lung (HandE staining), **(C)** CD68, **(D)** CD4, **(E)** PD-L1 staining, **(F)** PD-1 staining, **(G)** IFN-α in challenged lung, **(H)** IFN-α in control. White arrow shows a group of positive cells for each staining. Magnification are x20 for all representative images. *P*-value shows percentage differences of lung stained cells between challenged mice and controls (sample size 3 controls and 4 challenged mice).

Figure 3 shows mouse lungs developed non-caseating granuloma after MAB microparticles challenge. The inflammatory lesions were scored and showed significant increase in inflammation by H&E staining with marked increase in cells staining for macrophage marker, CD68, and PD-L1. IHC staining for IFN-α also showed significant increasing in bronchial cells in comparison with controls (*P* < 0.00001).

### IFN I Signaling Pathway Genes Are Overexpressed in Mouse Lungs Following Exposure to MAB

**Figure 4:**
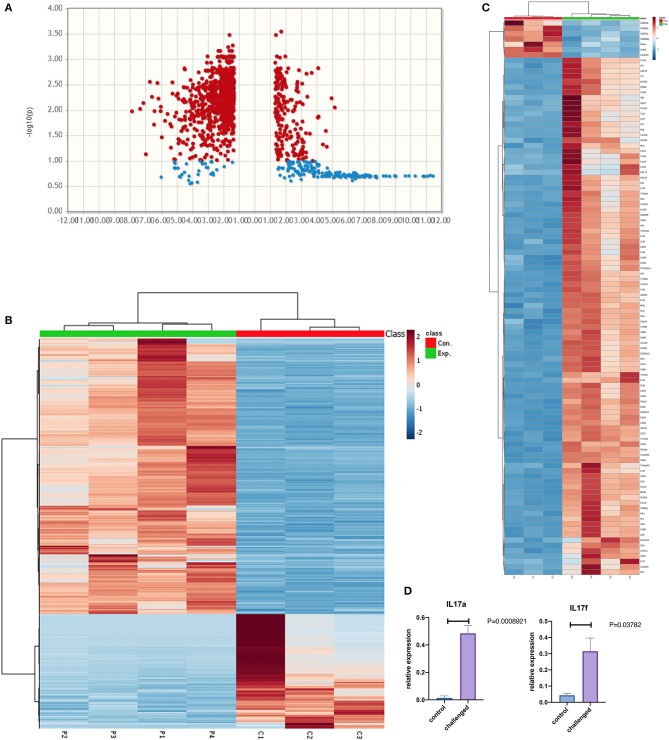
**(A)** Volcano and **(B)** heatmap for unsupervised clustering of the RNAseq transcriptomes according to pearson correlation. **(C)** Heatmap of immunogens, and **(D)** Lung *IL17a* and *IL-17f* gene expression in the lung of MAB microparticle challenged mice (sample size=controls and 4 challenged mice).

RNAseq analysis identified 1759 differentially expressed genes between NHBE cells challenged with and without MAB microparticles with FDR < 0.5, 1155 genes had a 2.5-fold change (FC) or greater. Volcano plots reveal differential expression of genes between NHBE cells with and without exposure to MAB microparticles (Figure 4A). Figure 4B shows the heatmap for unsupervised clustering of the RNAseq transcriptomes according to pearson correlation. Gene expression for each gene was normalized across samples to percentages ranging from marked upregulation (deep red) to marked downregulation (deep blue). Many immunogens were significantly upregulated in challenged mice by MAB microparticles (Figure 4C). Figure 4D shows significant upregulation of *IL-17a* and *IL-17f* genes in mice lung after exposure to microparticles.

Pathway enrichment analysis for selected genes with a FDR <0.05 differentially expressed between mice lung cells treated with saline (control) vs. MAB microparticles. MAB microparticle challenged lungs significantly showed gene pathway enrichment in the type I interferon pathway (GO:0071357) and type I IFN signaling pathway (GO:0060337) (adjusted *p* = 1.757e-19, and *p* = 8.783e-20, respectively). The top upregulated genes from the IFN I family were *IRF1* (FC:2.54), *IFIT3* (FC:2.55), *ISG15* (FC:2.56), *MXD3* (FC:3), *IRF8* (FC:3.05), and *MX1* (FC:3.10).

### IFN-α Proteins Overexpression in the Lungs Following Exposure to MAB

**Figure 5:**
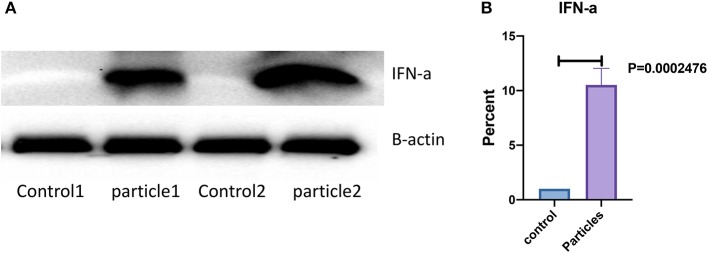
IFN-α protein expression was significantly increased in lung after MAB microparticle challenge. **(A)** Shows a representative image of western blot bands, **(B)** shows significant difference in IFN-α protein expression in the lung of challenged mice (sample size 4 for each group).

Expression of IFN-α in bronchial and granulomatous inflammatory cells were significantly increased following exposure to MAB cell wall microparticles (*P* < 0.00001). Western blot analysis of IFN-α protein expression found significant increase in microparticle challenged lung tissue (*P* = 0.0002) compared to negative controls (Figure 5).

## Discussion

This study found upregulation of 11 genes of the IFN 1 signaling pathway, upregulation of all 3 species of IL-36 (α, β, and γ) and upregulation of leukocyte chemokines in NHBE cells after exposure to microparticles of MAB. Mouse lungs challenged with MAB cell wall microparticles showed a granulomatous reaction with significant upregulation of IFN 1 genes. We also demonstrated that protein expression of MX1, OSA1, ISG15, and IFN-α were upregulated after MAB-host interaction in *in vitro* and *in vivo* models. *IL-17a* and *IL-17f* were upregulated in mice lung after exposure to microparticles. These data show MAB cell walls elicit a proinflammatory reaction from NHBE cells that likely initiates the host response to MAB infection. Finding similar gene expression changes in mice exposed to MAB particles confirms the bronchial epithelia response in an intact organism.

IFN I genes play an important role in controlling viral infections in bronchial epithelia and our study implicates their role in the host response to mycobacterial disease. IFIT1, ISG15, ISG20, and OAS 1,2, and 3 inhibit protein synthesis and cell proliferation in viral infected host cells. MX1 protein inhibits viral nucleoprotein synthesis and endocytosis (20). Given that mycobacteria are also intracellular pathogens, IFN response genes may also form the first layer of innate defense in upregulating macrophage and T cell specific genes including IL-17.

NHBE cells treated with MAB microparticles significantly upregulated all three subtypes of IL36 (α, β, and γ). IL36 belongs to the IL1 superfamily and is expressed by bronchial epithelial cells. IL-36 activates the pro-inflammatory transcriptional factor nuclear factor kappa B (NFκB), induces T Helper cell type 1 (Th1) responses by enhancing cell proliferation and IL2 secretion (21, 22) and is implicated in the inflammatory response from skin epithelial cells in psoriasis (23). IL36 is known to control IFN I related gene expression in a time dependent manner (24) and may play a role in NHBE response to MAB cell wall components. Our *in vitro* study also showed a significant upregulation in IL-36 genes and members of the *IL1* superfamily genes suggesting a possible link between IL-36 expression from NHBE cells leading to type I IFN gene expression via autocrine loop.

MAB exposed NHBE also produced chemokines *CCL5* and *CCL22* that are strong leukocyte chemoattractants. The gene of both chemokines were upregulated in the mice lung after challenging with MAB cell wall microparticle. CCL5 is a potent monocyte and macrophage attractant recruiting important immune cells to combat mycobacterial infections. The immune response to MAB is T cell dependent and that macrophages develop pathologic features of mycobacterial disease known as granulomas. We found significant granulomatous reaction in the lung of challenged mice that could suggest functional activity of upregulated *CCL5* and *CCL22*. Interestingly, MMP9 is a critical protein required to recruit macrophages and develop well organized granulomas in *M.TB* infections (25). Thus, NHBE expression of MMP may also initiate the granuloma formation commonly seen in mycobacterial infections.

These data strongly support the role of NHBE cells in the host defense against MAB infections. They suggest that bronchial epithelial cells play a central role in initiating an innate immune response producing the initial signal alerting resident macrophages to the site of infection and producing IL36 and type I IFN genes to add to the host defense. Furthermore, this study underscores the importance of mycobacterial cell wall antigens in initiating the innate immune response. Understanding the direct impact of the IFN I genes and IL36 production by NHBE cells during MAB infection will provide data to develop strategies to treat or prevent NTM infections.

## Data Availability Statement

The raw data supporting the conclusions of this manuscript will be made available by the authors, without undue reservation, to any qualified researcher.

## Ethics Statement

The studies involving human participants were reviewed and approved by University of Miami Ethic Committee. The patients/participants provided their written informed consent to participate in this study. The animal study was reviewed and approved by Miami VA Animal Care and Use Committee (IACUC).

## Author Contributions

CZ and NF performed experiments and helped in manuscript preparation. HA conducted literature review, helped to develop first draft of manuscript. GH and MC assisted with analyzing results and manuscript preparation. AG performed bioinformatic analysis and assisted in manuscript preparation. PB reviewed lung pathology slides. MM conducted literature review, conducted exploratory analysis, performed data analysis and pathway analysis, and manuscript preparation.

### Conflict of Interest

The authors declare that the research was conducted in the absence of any commercial or financial relationships that could be construed as a potential conflict of interest.

## Supplementary Material

Figure S1 Development of granulomas in mouse lung. Anesthetized 8 week old male, C57BI/6 mice **(A)** are injected with an 18-G IV catheter **(B)** to install *Mycobacterium abscessus* cell wall microparticles **(C)** into the lung **(D)**.

Figure S2 Shows injection of methylene blue in trachea using the method described in Figure S1. Lung shows blue color that confirms the correct technique.
