# Supplementary figures and images for: Mycobacterium abscessus—Bronchial Epithelial Cells Cross-Talk Through Type I Interferon Signaling
Source: Front Immunol. 2019 Dec 9;10:2888. doi: 10.3389/fimmu.2019.02888 (PMC6913194; doi:10.3389/fimmu.2019.02888)

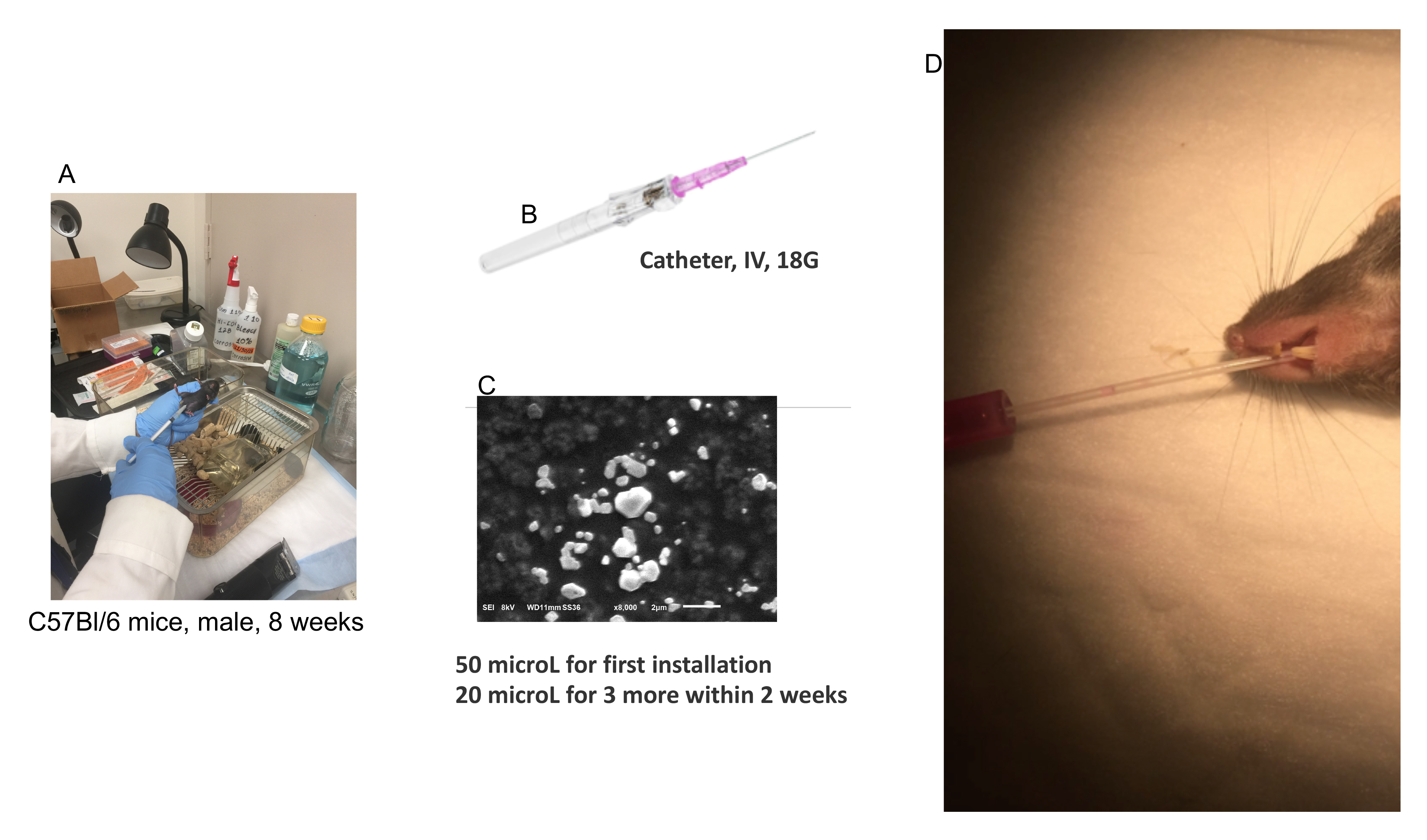

Supplement: Figure S1 — Development of granulomas in mouse lung. Anesthetized 8 week old male, C57BI/6 mice (A) are injected with an 18-G IV catheter (B) to install Mycobacterium abscessus cell wall microparticles (C) into the lung (D). [file Image_1.JPEG]

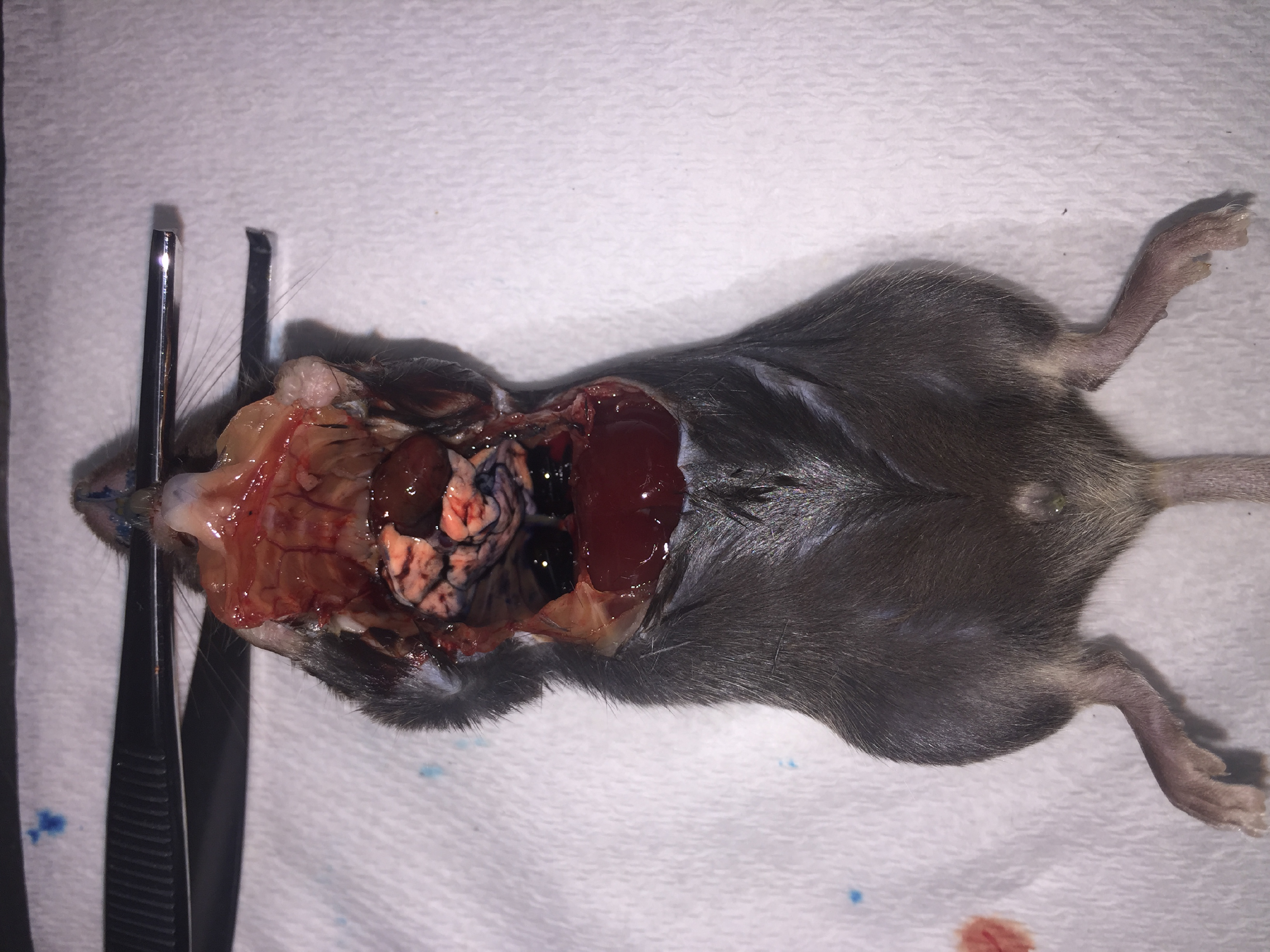

Supplement: Figure S2 — Shows injection of methylene blue in trachea using the method described in Figure S1. Lung shows blue color that confirms the correct technique. [file Image_2.JPEG]
